# Polymyxin B-associated hearing impairment in a patient with renal dysfunction- a case report

**DOI:** 10.1186/s12882-026-05120-8

**Published:** 2026-06-15

**Authors:** Xun Ni, Tingting Ding, Dandan Gong, Xiaojuan Liu, Jinna Wang, Huanhuan Wu, Xu Wang

**Affiliations:** 1 Department of Intensive Care Medicine, The Affiliated Suqian Hospital of Xuzhou Medical University, Suqian, China; 2 Department of Pharmacy, The Affiliated Suqian Hospital of Xuzhou Medical University, Suqian, China; 3 Nanjing Drum Tower Hospital Group Suqian Hospital, Suqian, China

**Keywords:** Polymyxin B, Drug adverse reaction, Neurotoxicity, Renal dysfunction, Hearing impairment

## Abstract

**Background:**

Polymyxin B (PMB) is a polypeptide antibiotic primarily used to treat infections caused by multidrug-resistant gram-negative bacteria. Its main adverse effects are neurotoxicity and nephrotoxicity. Neurotoxicity may manifest as irritability, fatigue, lethargy, ataxia, dysgeusia, limb numbness, and blurred vision. Hearing impairment attributed to PMB is rarely reported. Herein, we describe a case of hearing impairment in a patient with renal dysfunction, potentially related to intravenous PMB therapy.

**Case presentation:**

A 54-year-old, 70-kg male with poorly controlled type 2 diabetes (complicated by nephropathy, neuropathy, retinopathy), end-stage kidney disease (on hemodialysis), and hypertension was admitted to the ICU for gastrointestinal bleeding and hemorrhagic shock. On postoperative day 7, he developed carbapenem-resistant Acinetobacter baumannii (CR-AB) pneumonia and was treated with PMB 50 mg q12h plus sulbactam 2 g q8h. Therapeutic drug monitoring (TDM) showed the area under the plasma concentration-time curve across 24 h at steady state (AUC_ss, 24 h_) of PMB was 37.7 mg·h/L. PMB was increased to 100 mg q12h, after which the patient reported hearing impairment, with a corresponding AUC_ss, 24 h_ of 152.8 mg·h/L. The dose was then reduced and subsequently discontinued. The patient’s hearing fully recovered one day after discontinuation. No formal audiologic testing was performed; the diagnosis relied on patient self-report and clinical observation.

**Conclusions:**

This case highlights the importance of recognizing neurotoxic events related to PMB, including hearing impairment. Close monitoring and timely intervention are especially critical in patients with renal insufficiency or those receiving high-dose PMB. Accordingly, TDM plays an important role in anticipating and avoiding such adverse reactions.

## Background

Polymyxin B (PMB) is a polypeptide antibiotic that exerts bactericidal effects primarily by disrupting the cell membrane integrity of gram-negative bacteria [1, 2]. Due to its activity against gram-negative bacteria, PMB has re-emerged in clinical practice as a key agent against multidrug-resistant infections [3–5]. However, its clinical use is limited by significant adverse effects, notably neurotoxicity and nephrotoxicity. Documented adverse events include bleeding, respiratory paralysis, rhabdomyolysis, skin hyperpigmentation, and acute kidney injury [6–14]. Hearing impairment attributed to PMB has rarely been reported. Herein, we present a case of hearing impairment possibly associated with elevated PMB concentrations.

## Case presentation

A 54-year-old male weighing approximately 70 kg was admitted to the intensive care unit (ICU) due to gastrointestinal bleeding and hemorrhagic shock on April 6, 2023. He had a 5-year history of hypertension and an 18-year history of poorly controlled type 2 diabetes, with complications including diabetic nephropathy, peripheral neuropathy, and retinopathy. One year prior, he was diagnosed with end-stage renal disease (ESRD) due to anasarca and has been hospitalized several times for this condition.

**Fig. 1:**
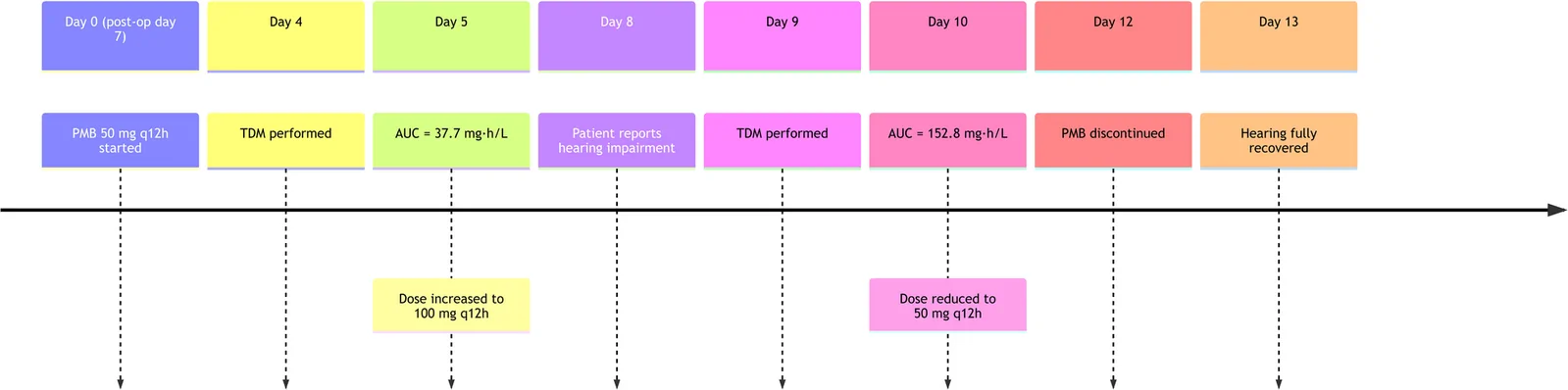
A timeline of PMB dosing, TDM measurements, symptom onset, and recovery

During initial hospitalization, exploratory laparotomy revealed a duodenal ulcer with massive hemorrhage. And the patient was treated with renal replacement treatment (RRT) intermittently due to ESRD during hospitalization. On postoperative day 7, the patient developed hospital-acquired pneumonia, with fever (peak 38.5 °C), purulent endotracheal secretions, and sputum culture confirmed carbapenem-resistant *Acinetobacter baumannii* (CR-AB). As shown in Fig. 1, targeted therapy was initiated with intravenous PMB 50 mg q12h combined with sulbactam 2 g q8h. Therapeutic drug monitoring (TDM) performed after the seventh dose showed peak (*C*_max_)and trough (*C*_trough_) concentrations of 2.52 and 0.88 mg/L, respectively. Using standard equations [15], the area under the plasma concentration-time curve across 24 h at steady state (AUC_ss, 24 h_) was approximately 37.7 mg·h/L, below the recommended target range of 50–100 mg·h/L [15]. As the patient remained febrile with persistent secretions and atelectasis confirmed by chest computed tomography (CT), the PMB dose was increased to 100 mg q12h. Two days later, the patient reported hearing impairment. No formal audiologic assessment was conducted, but TDM performed after the sixth dose of the adjusted regimen showed *C*_max_ and *C*_trough_ concentrations of 8.34 and 4.71 mg/L, respectively, with an AUC_ss, 24 h_ of 152.8 mg·h/L, exceeding the recommended upper threshold [15, 16]. Consequently, the PMB dose was reduced to 50 mg q12h. After one additional day, sputum culture was negative for CR-AB, and body temperature was 37.3 °C, prompting discontinuation of PMB. One day after discontinuation, the patient reported normal hearing. Following three more days of continued observation, the patient’s condition stabilized, and he was transferred to the nephrology department for further management.

## Discussion

This case describes hearing impairment potentially associated with PMB neurotoxicity. The reported incidence of polymyxin-related neurotoxicity varies widely. Earlier studies reported incidences of 27% and 7.3% for intravenous and intramuscular colistimethate, respectively [17, 18]. A recent study in renal transplant patients found a neurotoxicity incidence of 63.4% [8], while in healthy volunteers, it was as high as 85% [19]. Few reports specifically address PMB-associated hearing impairment. Several factors may explain this underrecognition. First, neurotoxicity has likely been underestimated due to a predominant focus on nephrotoxicity. Second, many studies are retrospective, and neurotoxicity symptoms may not have been consistently recorded [14, 19, 20]. Third, there is no objective test for neurotoxicity analogous to creatinine clearance for nephrotoxicity, and assessment relies heavily on patient self-report, which may be compromised in critically ill or intubated patients. In healthy volunteers, higher reported incidence may reflect their ability to articulate symptoms [19, 20].

The mechanism of PMB-induced neurotoxicity remains unclear. Hypotheses include neuromuscular blockade due to interactions between polymyxins and high-lipid-content neurons, blocking acetylcholine release [21]; prolonged depolarization associated with calcium depletion [22]; and oxidative stress-induced apoptosis with excessive reactive oxygen species (ROS) leading to mitochondrial damage [23–25]. A recent animal study also suggested that PMB may impair the blood-brain barrier [26]. Additionally, neurotoxicity may be dose-related [19, 22].

In this case, the patient received PMB plus sulbactam for CR-AB pneumonia. After dose escalation, the patient reported hearing impairment, with an AUC_ss, 24 h_ of 152.8 mg·h/L. Notably, current guidelines define toxicity thresholds primarily for nephrotoxicity (e.g., AUC > 100 mg·h/L associated with increased AKI risk) [15]. A defined threshold for ototoxicity is not yet established, and the relationship between PMB exposure and hearing impairment remains uncertain. Therefore, the association observed here should be interpreted cautiously. Following dose reduction and discontinuation, hearing recovered completely within one day. This rapid reversibility is atypical for drug-induced ototoxicity, which is often irreversible or recovers slowly [27]. Possible explanations include reversible neurodysfunction rather than permanent cochlear damage, transient inner ear ionic or hemodynamic changes, or reporting bias. This further underscores the need for objective audiologic assessment in future cases.

Alternative explanations for the patient’s hearing symptoms include uremic neuropathy, ICU-related encephalopathy, concomitant medications (e.g., sedatives, other potentially ototoxic drugs such as diuretics or vancomycin), or hemodynamic instability. However, during the period between the medication adjustment and the onset of hearing impairment, the patient did not take any analgesics, sedatives or vancomycin. And there were no other cranial nerve abnormalities or limb paresthesia. Furthermore, neither clinical manifestations nor laboratory findings indicated any deterioration of the patient’s condition. The symptom resolved specifically after PMB discontinuation, supporting a causal role. Nevertheless, these competing causes cannot be fully excluded, especially in the absence of formal audiometry.

Regarding antimicrobial strategy, the decision to escalate PMB rather than switch to alternative agents was based on limited therapeutic options for CR-AB, the patient’s clinical instability, and the lack of alternative agents with reliable activity against the isolate. No other effective agents (e.g., tigecycline, cefiderocol) were available at the time. The dose increase was considered despite ESRD because PMB is primarily eliminated via non-renal pathways [16]. In retrospect, closer TDM monitoring and earlier dose adjustment might have prevented supratherapeutic exposure.

## Conclusion

This report highlights the importance of recognizing PMB-associated neurotoxicity, including hearing impairment. Close monitoring for adverse effects and timely intervention are essential, especially in patients with renal insufficiency or those receiving high doses. Furthermore, TDM is a valuable tool for predicting and preventing adverse reactions.

## Data Availability

The datasets generated during and/or analyzed during the current study are available from the corresponding author on reasonable request.

## Abbreviations

PMB: Polymyxin B
ICU: Intensive care unit
ESRD: End-stage renal disease
RRT: Renal replacement treatment
CR-AB: Carbapenem-resistant Acinetobacter baumannii
TDM: Therapeutic Drug Monitoring
*C*_max_: Peak concentration
*C*_trough_: Trough concentration
AUC_ss, 24h_: The area under the plasma concentration-time curve across 24 h at steady state
CT: Computed tomography
ROS: Reactive oxygen species
